# Noninvasive approaches to detect methylation-based markers to monitor gliomas

**DOI:** 10.1093/noajnl/vdac021

**Published:** 2022-11-11

**Authors:** Houtan Noushmehr, Grayson Herrgott, Natalia S Morosini, Ana Valeria Castro

**Affiliations:** Department of Neurosurgery, Omics Laboratory, Henry Ford Health System, Detroit, Michigan, USA; Department of Neurosurgery, Omics Laboratory, Henry Ford Health System, Detroit, Michigan, USA; Department of Neurosurgery, Omics Laboratory, Henry Ford Health System, Detroit, Michigan, USA; Department of Neurosurgery, Omics Laboratory, Henry Ford Health System, Detroit, Michigan, USA

**Keywords:** epigenetics, glioma, machine-learning, liquid biopsy, non-invasive biomarkers

## Abstract

In this review, we summarize the current approaches used to detect glioma tissue-derived DNA methylation markers in liquid biopsy specimens with the aim to diagnose, prognosticate and potentially track treatment response and evolution of patients with gliomas.

## Overview

Malignant transformation and/or therapeutic resistance causes pervasive relapse of glioma subtypes, leading to high death rates after the current treatment protocols (maximum surgical excision followed by chemoradiotherapy).^1^ Longitudinal monitoring of glioma evolution is currently performed through serial clinical and conventional magnetic resonance imaging (MRI) protocols.^2^ However, conventional or advancing imaging techniques, in addition to being costly and not available or feasible for some patients, this approach lacks the sensitivity or specificity required to diagnose minimal residual tumor burden or discriminate true recurrence from necrosis or pseudoprogression.^2–6^ The final diagnosis of true progression, for instance, relies on histological assessment of tumor tissue obtained through surgical interventions which may not be clinically viable for some patients, resulting in potential postponements in the administration of salvage therapies.^7^ Furthermore, serial surgical procedures are not suitable for the detection of glioma heterogeneity or surveillance of the dynamic morphological and molecular changes frequently encountered during glioma evolution^8–10^.

Liquid biopsy has emerged as an attractive non- or minimally invasive approach to complement and overcome some of the challenges and limitations associated with surgical and imaging procedures in real-time detection and monitoring of glioma evolution.^11–13^ This approach involves the detection of tumor cells, extracellular vesicles (e.g. exosomes), and molecular elements fragments (e.g. nucleic acids) released by tumors into biofluids such as blood, cerebrospinal fluid (CSF), and urine.^11^ Cell-free DNA (cfDNA), particularly the tumor fraction (ctDNA), one of the molecular elements most frequently assessed in liquid biopsy specimens, is amenable to quantitative analysis and molecular interrogation through different epi(genetic) survey platforms.^11,12,14^

Among the epigenetic mechanisms that play key roles in glioma development and progression, DNA methylation abnormalities are the most frequently reported and are readily detected in liquid biopsy specimens.^12,14–16^ Interrogation of DNA methylation abnormalities using liquid biopsy specimens is advantageous in gliomas and other CNS tumors as they are pervasive and recurrent within tumor types, affect the entire genome, allow for the detection of methylation patterns or markers that backtrack to the cell-of-origin and highlight potential therapeutic targets, thus outperforming the profiling of somatic mutation sequencing for the diagnosing and monitoring of cancer.^16–22^ In the present review, we summarize the current approaches used to detect glioma tissue-derived DNA methylation markers in liquid biopsy specimens with the aims to diagnose, prognosticate and potentially track treatment response and glioma evolution/progression.

## DNA Methylation-Based Classifiers Using Glioma Tissue and Liquid Biopsy Specimens

### Tissue Specimens

Since 2016, the World Health Organization (WHO) recommended integration of molecular features to the classification of CNS tumors such as the mutational status of the isocitrate dehydrogenase (IDH) genes to segregate gliomas into IDH-mutant and IDH-wildtype subtypes, which present prognostic value.^23^ Interestingly, IDH-mutant gliomas manifest the cytosine-phosphate-guanine (CpG) island methylator phenotype (G-CIMP) and have generally more favorable prognosis than their IDH-wildtype counterparts.^16,24,25^ IDH-mutants may be further stratified into G-CIMP-high and G-CIMP-low subsets according to higher or lower genome-wide methylation levels, respectively.^16,24,26,27^ Unlike patients with G-CIMP-high gliomas, those affected by G-CIMP-low tumors have a poorer prognosis and lower methylome levels, which are also observed in patients carrying IDH-wildtype gliomas.^16,24,26,27^ Studies involving the longitudinal analysis of tissue methylomes observed a subset of G-CIMP-high glioma which loses methylation at recurrence and acquires a G-CIMP-low phenotype associated with a more aggressive behavior.^26,27^ Comparison of the methylomes of G-CIMP-high gliomas which progress to G-CIMP-low phenotype to those which retain their high methylation status at first recurrence yielded the identification of a set of methylation signatures able to predict risk of progression to a more aggressive subtype across independent cohorts (namely glioma prognostic classifier).^26,27^ In a recent longitudinal study involving homogeneously treated IDH-mutant anaplastic astrocytomas, now referred to as Astrocytoma, IDH-mutant, WHO Grade 3, the authors confirmed that patients classified as G-CIMP-low at diagnosis or recurrence presented worse overall survival than their G-CIMP-high counterparts.^27^

Analysis of tissue methylomes across low- and high-grade diffuse gliomas led to the identification of DNA methylation groups presenting distinct clinicopathological, genomic, and prognostic features.^15,16,24^ The identification of methylation signatures specific to each subtype allowed for the development of a machine learning-based model, namely the glioma methylation subtype classifier, which exhibited high accuracy in stratifying an independent cohort according to specific methylation groups.^16,26,27^

Given that genome-wide DNA tissue methylation patterns are conserved across cell and tumor types that originate from a common lineage, the profiling of this molecular feature constitutes a reliable and reproducible approach to detect methylation fingerprints to diagnose and classify tumors, including those of the CNS.^12,13,20,28–30^ Based on CNS tumor tissue methylome signatures, a machine learning-based classifier was able to, with high accuracy, discriminate across and within tumor types, including gliomas, and has been incorporated into the 2021 edition of the World Health Organization CNS tumor classification.^27,29,30^ The use of this DNA methylation-based classifier has shown to be especially advantageous in distinguishing tumors with unusual histopathological features or specimens with small amounts of tumor.^30,31^ In summary, detection of glioma-relevant DNA methylation markers has the potential to change diagnostic, prognostic, and patient-monitoring paradigms.

## Methylation Markers in Glioma Liquid Biopsy Specimens

Studies have shown that methylation profiling of cell-free DNA released from CNS tumors in blood (serum or plasma) and other biosources (circulating tumor cells, extracellular vesicles, urine and CSF) allows for the detection of tumor-specific molecular markers^11–14,28^ (Tables 1 and 3).

**Table 1.** Description of Studies Involving Liquid Biopsy-Based Detection of Molecular Markers in Gliomas

Experimental Assay						Studies			
Molecular Sources			**Assay**	**Biosource**	**Purpose**	**Experimental Design**	**Conclusion**	**Clinical Value**	**Reference**
Genome Wide		**CTC counting**	Ficoll-Paque	Whole Blood	Identification of stemness properties in GBM-derived CTCs	Cross-sectional	GBM-derived CTCs express stemness properties	Diagnosis/ Prognosis/ Monitoring	Liu et al., 2018
		**Single-Cell**	scRNA-seq						
	**cfDNA profiling**	**DNA methylation**	cfMeDip-Seq	CSF, Serum	Detect methylation patterns in promoters of MGMT, p16INK4a, TIMP-3 and THBS1	Cross-sectional	Promoters present a hypermethylation pattern that can be accessed with 100% specificity in tumor tissues, serum and CSF	Prognosis	Liu et al., 2010
Targeted	**cfDNA profiling**		MSP	Serum	Investigate the promoter DNA methylation patterns in matching glioma tissue and serum	Cross-sectional	Methylation profile of *MGMT, p16, DAPK*, and *RASSF1A* gene promoters in GBM-derived serum DNA corresponds to matching tissue	Prognosis/ Theranostic	Balaña et al., 2003
Targeted				Serum	Evaluate MGMT, p16INK4a, TIMP-3 and THBS1 gene promoter hypermethylation	Cross-sectional	*MGMT* promoter methylation is likely associates wih heterogeneity, aggressiveness and evolution	Prognosis	Lavon et al., 2010
	**cfDNA profiling**	**DNA methylation**	PYR	Whole Blood	Characterize MGMT methylation status and concordance across paired blood and glioblastoma tissue performing MSP and PYR approaches	Cross-sectional	Both approaches showed less sensitivity (average sensitivity of 31.5%) in capturing methylation marks in cfDNA than in glioblastoma tissue samples. The specificity of MSP assay in blood reached a value of 96% while the PYR method presented a value of 76% in plasma.	Prognosis	Estival et al., 2019
			Methyl-BEAMing	Plasma	Evaluate MGMT promoter methylation status using plasma cfDNA from patients with GBM	Cross-sectional	Methyl-BEAMing assay is applicable to different specimens such as FFPE samples with good sensitivity, specificity and reproducibility	Prognosis	Barault et al., 2015
Targeted		**CTC counting**	CTC-iChip	Whole Blood	Elucidate the metastatic profile of the GBM cells	Cross-sectional	GBM cells harbor mesenchymal features that confer an invasive ability within the brain but no metastatic strength and power	Diagnosis/ Prognosis	Sullivan et al., 2014
			SE-iFISH	Whole Blood	Investigate GBM-derived CTCs as an approach to monitor glioma progression	Cross-sectional	The SE-iFISH was efficient and reliable to monitor the microenvironment of gliomas and to identify CTCs in the PB of 77% of patients affected by glioma	Prognosis/ Monitoring	Gao et al., 2016
	**cfDNA profiling**	**DNA fragmentation**	Eletrophoresis	Plasma	Identify the feasibility of using GBM-derived cfDNA in assessing glioma progression	Cross-sectional/ Longitudinal	Monitoring GBM-derived cfDNA levels in blood samples before diagnosis and during treatment surveillance (until progression) seems reliable	Prognosis/ Monitoring	Nørøxe et al. 2019

Notes: CTC, circulating tumor cells; CSF, cerebrospinal fluid; GBM, glioblastoma; PB, peripheral blood; OS, overall survival; FFPE, formalin-fixed paraffin-embedded; MSP, Methylated specific PCR; PYR, pyrosequencing; cfMeDIP-seq, cell-free methylated DNA immunoprecipitation-sequencing; SE-iFISH, subtraction enrichment and immunostaining-fluorescence *in situ* hybridization.

One of the main hindrances to the application of liquid biopsy in the management of patients with CNS tumors, particularly gliomas, is the minute amount of cellular and molecular elements released by these tumors into biofluids and the obtention of good quality DNA for downstream molecular profiling. Currently, there are no standardized methodologies to detect and profile these molecular elements, and the sensitivity of current methods is highly variable.^11,14,32,33^ However, ongoing technological advances show promise in improving the accuracy of these methods^11–14,34,35^ (Tables 1 and 3). A summary of studies reporting on the application of DNA methylation-based liquid biopsy studies in patients with gliomas is displayed in Tables 1 and 3.

## Assessment Approaches for cf- and ctDNA Analysis

Circulating cell-free DNA (cfDNA) originates from healthy and neoplastic cells as a result of necrosis, apoptosis of nucleated cells, and/or active secretion.^36^ Interestingly, measurement of total plasma cfDNA concentration, i.e., a combination of both tumor- and nontumor-derived cfDNA, has shown clinical relevance. For instance, studies revealed that patients with high-grade glioma had significantly higher plasma cfDNA concentration compared to patients with low-grade glioma at initial diagnosis or healthy controls.^37,38^ In a pilot prospective study,^37^ the authors showed that the pre-surgical concentration of glioblastoma-derived plasma cfDNA was associated with lower progression-free survival rates (PFS); additionally, the cfDNA level correlated with radiological tumor burden and increased during tumor progression, after radiation therapy.

An important challenge in the molecular downstream analysis of total cfDNA is discriminating between ctDNA signals from the nontumor-derived cfDNA.^14,39^ In response, several strategies to enhance the detection sensitivity of the ctDNA fraction in plasma or serum have been developed such as the detection of knowingly tumor-specific gene mutations, copy number alterations or methylation signatures characteristic of each tumor type in cfDNA specimens^14,19,22,40^ or the profiling of cfDNA fragments (fragmentomics) to select lengths characteristic of ctDNA in plasma, urine, or CSF. The latter may be followed by downstream genomic or epigenomic molecular analysis as described in gliomas and other tumors.^39,41–43^

## cfDNA Methylation Profiling Methods

The description, advantages, and limitations of DNA methylation-based and other methods applied to liquid biopsy specimens is reviewed elsewhere^14,39^ and summarized in Table 2.

**Table 2.** Summary of Assays and Methodologies Applied to Different Molecular Sources of Liquid Biopsy Specimen

*Molecular Sources*		*Assay*	*Methodology*	*Description*	*Advantages*	*Limitations*
	***Extracellular Vesicles (EVs)***	Next-generation sequencing	Sequencing	Based on next-generation sequencing	EVs have the ability to cross the BBB and reach the bloodstream, harboring oncogenic molecular repertoire which includes nucleic acids, proteins and tumor metabolites	Glioma EVs represent only almost 10% of the total EV found in patient plasma hampering the sensitivity of all the analysis
		RT-PCR	Real-time PCR	Based on quantitative real-time PCR		
		Flow cytometry		Based on the detection and measurement of physicochemical properties of cells or molecules		
		Mass spectrometry		Based on the measurement of the mass-to-charge ratio (m/z) of different molecules		
***cfDNA profiling***	***DNA Fragmentation***	Whole genome sequencing (WGS)	Sequencing	Sequencing of the genome	Expensive	(*i*) Low sensitivity (5–10%); (*ii*) cfDNA concentration-dependent
		Whole exome sequencing (WES)	Sequencing	Sequencing of the protein-coding regions		
		q-PCR	Amplification of genetic sites	Based on the amplification and quantification of a specific DNA sequence	Low cost	Requires tumor-derived signatures prior to analysis
	***DNA Methylation***	Methyl-specific PCR (MSP)	Bisulfite conversion + PCR	Based on the combination of RRBS and PCR amplification	(*i*) High sensitivity and specificity; (*ii*) Requires low DNA input; (*iii*) Relative measurement of target region	(*i*) Degrades the DNA molecule; (*ii*) Does not provide quantitative assessment of the methylation profile; (*iii*) PCR bias
		Methyl-BEAMing	PCR	Based on the combination of ddPCR and fluorescent hybridization probes (flow cytometry)	(*i*) High sensitivity and specificity; (*ii*) Requires low DNA input; (*iii*) Relative measurement of target region	(i) Inaccurate; (*ii*) Requires primer design; (*iii*) Depends on DNA input
		Droplet Digital PCR (ddPCR)	PCR	Based on droplet digital PCR		
		Pyrosequencing (PYR)	Sequencing	Based on incorporation of PPi into the nucleotide during PCR process	Estimates the methylation signal of regions containing both and increase and a reduced level of methylation	(*i*) Estimates the methylation signal only from short regions (~350bp); (*ii*) Time-consuming; (*iii*) Labor-intensive steps
		HM850 – EPIC platform	Array	Detection and measurement of selected methylation CpG sites across the genome	(*i*) Easy to use; (*ii*) Time-efficient; (*iii*) Cost-effective; (*iv*) High-throughput capabilities	(i) Require significant amount of DNA; (*ii*) Do not provide a comprehensive methylation profile—low coverage of intergenic regions; (*iii*) DNA degradation
		HM450- 450k platform	Array		(*i*) Does not require PCR; (*ii*) allows integration with RNA-seq data	
		Next Generation Sequencing (NSG)	Sequencing	Based on next-generation sequencing	(*i*) Detects new genetic mutations; (*ii*) High multiplexing capability	(*i*) Expensive; (*ii*) Time-consuming; (*iii*) Inability to perform longitudinal tumor evaluation
		cfMeDIP-Seq	Immunoprecipitation	Based on detection of cell-free methylated DNA by immunoprecipitation and high-throughput sequencing	(*i*) Sensitive; (*ii*) Cost-efficient; (*iii*) Bisulfite-free	(*i*) Relies on mapping of reads at single CpG loci; (*ii*) Does not detect unmethylated signals
	***5hmC***	5hmC-Seal	Chemical reaction	Based on profiling the spatial and temporal distribution of 5hmC by using a T4 bacteriophage β-glucosyltransferase to induce a chemical reaction between an azide group and the hydroxyl group of 5hmC	(*i*) Efficient in working with low amounts of DNA (about 5 ng); (*ii*) Unbiased approach	Low resolution
	***Cell tumor cells (CTC) Counting***	CellSearch®	Antibody epitope selection	Cell selection based on antibody-based beads	Approved by FDA	Low sensitivity
		CTC-iChip		Cell selection by magnetically tagged antibodies and hydrodynamic size-based cell separation	High efficiency	Low reproducibility due to tumor gene expression disarrangement
		Parsortix™	Spiral microfluidic device	Based on the detection of CTCs through their size, rigidity, deformability and compressibility	(*i*) Does not require pre-processing; (*ii*) Sensitive in capturing single and rare CTCs; (*iii*) Detects EpCAM-negative cells; (*iv*) Fast and inexpensive	(*i*) This system is time-consuming (~ 7 hours); (*ii*) not approved by FDA
		SE-iFISH	Fluorescence Immunocytochemistry	Cell selection based on using antibody-based fluorescent probes	(*i*) Low cost; (*ii*) High sensitivity	(*i*) Low sensitivity; (*ii*) imprecise and biased; (*iii*) Lack of standardization and reproducibility
		Ficoll-Paque	Density gradient centrifugation	Cell separation by specific layer separation using synthetic polymer of sucrose (Ficoll PM400) and epichlorohydrin andsodium diatrizoate	(*i*) Inexpensive; (*ii*) Reliable	(*i*) Low efficiency in isolating large CTCs and clusters; (*ii*) Low purity
	***Single-Cell***	scRNA-seq	Sequencing	Based on isolation and library construction of individual transcriptional landscapes	High sensitivity to detect gene expression levels and new transcripts	(*i*) Expensive; (*ii*) Laborious steps; (*iii*) Data bias
		scATAC-seq	Sequencing	Based on isolation and library construction of individual transcriptional landscapes.	High specificity to detect regulatory regions and elements	(*i*) Sparsity of signals; (*ii*) Low detection efficiency
		Motorized approach	Micromanipulation	Manual selection of cells based on the integration between inverted microscopy and joystick controller	High sensitivity	(*i*) Time-consuming; (*ii*) Error-prone; (*iii*) Labor- intensive
		Digital droplet PCR (ddPCR)	Microfluidic system	Sample is partitioned into individual droplets prior to their amplification and fluorescence detection	(*i*) Efficient in capturing small cell droplets; (*ii*) Simple and fast protocol; (*iii*) Low cost	Inability to remove rRNA ribosomal RNA

Note: FDA, Food and Drug Administration (U.S); BBB, blood–brain barrier; RRBS, reduced representation bisulfite sequencing; ddPCR, droplet digital PCR; PPi, inorganic pyrophosphate; 5hmC, 5-hydroxymethylcytosine.

**Table 3.** Machine Learning-Based Classifiers Using Tissue and Liquid Biopsy Specimens From Patients with Gliomas

*Sample Source*	*Molecular marker of Interest*	*Profiling Method*	*Objective*	*Comparison* *Groups (n)*	*Study Design*	*ML Algorithm*	*Feature* *Selection*	*Cohort Sampling*	*Model Performance*	*Clinical Applications*	*Reference*
***Liquid Biopsy Serum***	CpG Methylation	Microarray (EPIC)	Application of serum-based cfDNA methylation markers to diagnose and prognosticate gliomas	Gliomas [*n* = 96] vs CNS tumors and controls [*n* = 112].	Cross Sectionaland Longitudinal	RF	Glioma- specific differentially methylated probes (*n* = 500)	Holdout Method & Tenfold Cross Validation	ACC: 0.99 [SE: 1.00; SP: 0.98%]	Diagnostic & Prognostic	Sabedot et al., 2021
***Liquid Biopsy*** ***Plasma***	CpG Methylation	cfMeDip seq	Plasma cfMeDIP-seq performance to detect and differentiate across brain tumor types	Diffuse gliomas [*n* = 122] vs other CNS tumors and controls[*n* = 98].	Cross- sectional	RF	Differentially methylated regions (*n* = 300)	Holdout Method	AUC = 0.99 [95% (CI: 0.96–1.0)]	Diagnostic	Nassiri et al., 2020
	5-hydroxymethylcytosine (5hmC)	5hmC- Seal	Develop a noninvasive diagnostic approach for gliomas using plasma cfDNA	Gliomas[ *n* = 111] vs controls [*n* = 111]; LGG [*n =* 47] vs GBM [*n* = 64].	Cross- sectional	GLM	Differential 5hmC modification distribution	Holdout Method & Fivefold Cross Validation	AUC = 0.88; [95% (CI: 0.77–1.00)]	Diagnostic	Cai et al., 2021
	ctDNA Fragmentation	Shallow whole Genome Sequencing	Determine, compare and characterize the presence of cfDNA in biofluids	Pan-cancer, including glioma [*n* = 12] vs controls[*n* = 8].	Cross-sectional	LR & RF	5 variables	Holdout Method & Fivefold Cross Validation	LR and RF AUC = 0.9	Diagnostic	Mouliere et al., 2018
***Liquid Biopsy*** ***Urine***	ctDNA Fragmentation	Shallow whole Genome Sequencing	Determine the glioma-derived DNA fragments in CSF, plasma and urine samples using a personalized approach	glioma [*n* = 35] vs controls[*n* = 53]	Cross-sectional	LR, RF, SVM, GLMEN	10 size features	Holdout Method & Fivefold Cross Validation	LR and RF AUC = 0.9 SVM: AUC = 0.8 GLMEN: AUC = 0.91	Diagnostic	Mouliere et al., 2021
***Tumor Tissue***	CpG Methylation	Microarray (450k)	Comprehensive molecular profiling of low- and high-grade glioma	Gioma (LGG and HGG) [*n* = 1122]	Cross-sectional	RF	IDH-mutant tumor-specific (*n* = 1308), mutant subtypes (*n* = 163), and wild-type-specific (*n* = 914).	Not reported	Accuracy:88%	Molecular subtyping classifier)	Ceccarelli et al.
	CpG Methylation	Microarray (EPIC)	Identify DNA methylation markers in G-CIMP samples associated with progression at follow-up	IDH-mutant G-CIMP-high [*n =* 132] vs G-CIMP-low[ *n* = 19]	Cross-sectional/ Longitudinal		Glioma methylation subtype classifier prognostic -methylation score (*n* = 7 probes)	Holdout Method	Average ACC > 0.95	Prognostic (glioma prognostic classifier)	Souza et al., 2017
	CpG Methylation	Microarray (EPIC)	Design a comprehensive approach for the DNA methylation-based classification of CNS tumors	CNS tumors [*n* = 2802]	Cross-sectional	RF [additional Multinomial LR]	Highest variant probes across tumor types (*n* = 10 000 probes).	Threefold Nested Cross Validation	AUC = 0.999[SE: 0.989; SP: 1.00)]	Diagnostic (CNS classifier)	Capper et al., 2018
	CpG Methylation	Microarray (EPIC)	Test and evaluate prognostic genetic and epigenetic biomarkers using the CATNON trial cohort	IDH-mutant anaplastic astrocytomas[n = 432]	Longitudinal	RF	Differential methylated probes	CNS and glioma prognostic classifiers	See Ceccarelli et al., Capper et al.	Prognostic	Tesileanu et al., 2021

Note: RF, Random Forest; LR, Logistic Regression; 5hmC-Seal, 5-hydroxymethylcytosine selective labeling; GLM, Generalized Linear Model; SVM, Support Vector Machine; GLMEN, Generalized Linear Model w/ Elastic-net Regularization; cfMeDIP-seq, cell-free methylated DNA immunoprecipitation-sequencing; cfMeDIP-seq, cell-free methylated DNA immunoprecipitation-sequencing.

### 5-Methylcytosine (5mc) and 5-Hydroxymethylcytosine (5hmC) Modification Profiling

The screening of 5-methylcytosine (5mc) and 5-hydroxymethylcytosine (5hmC, a demethylation marker resulting from 5mC oxidation) in liquid biopsy specimens holds great potential for early detection, diagnosis, prognosis, and monitoring of the dynamic changes and treatment outcomes in cancers, specifically gliomas^38,44–48^

High-throughput genome-wide (whole genome bisulfite sequencing [WGBS], reduced representation bisulfite sequencing [RRBS], sequence only genomic regions rich in CpG dinucleotides), and microarray assays are the most commonly reported sequencing methods to profile genomic or liquid biopsy-derived DNA. These methods indistinctly detect both 5mC and 5hmC modifications; however, adjustments such as simultaneous bisulfite and oxidative bisulfite treatments allow their resolution.^14,48^ Bisulfite-based methods provoke a significant degradation of DNA (over 90%) which may be detrimental to the detection of the low-input cfDNA amount released by CNS tumors.^14,40^ Adaptations of these methods or bisulfite-free sequencing protocols (e.g. chemical- or enzymatic-based and affinity enrichment methods) were developed to circumvent this limitation and to improve the detection of low-input DNA from tissue and liquid biopsy specimens.^14,48^ The methylated DNA immunoprecipitation (MeDIP) approach, for instance, is based on affinity enrichment with 5-methylcytosine (5mC) or 5hmC-antibodies followed by high-throughput sequencing of genomic (tissue) or cfDNA, namely MeDIP (Methylated DNA Immunoprecipitation) and cfMeDIP-seq, respectively and is efficient to detect small amount of DNA in tissue or liquid biopsy specimens. The application of cfMeDIP-seq to plasma samples was able to detect and discriminate across intracranial tumors, including gliomas.^13,28^

Another approach that has shown diagnostic value in gliomas is the specific profiling of the levels of genome-wide 5hmc, a cytosine modification with gene transcription regulatory role and tissue-specificity associated with the diagnosis and prognostication of many tumors.^38,49,50^ In a study, genome-wide 5hmc profiling using a highly sensitive and robust chemical labeling technique (5hmC-Seal technique) in plasma samples from patients with gliomas showed that 5hmc levels were higher in gliomas compared to healthy controls and several machine learning-based models using 5hmc concentration as input were able to discriminate gliomas from healthy controls and glioblastomas (grade 4).^38^

### Methylated Glioma-Specific DNA Detection

The feasibility of detecting methylated genes presenting biological or clinical relevance using targeted methods (methyl-specific or droplet digital PCR) or multiple regions of interest (target sequencing) in cfDNA specimens has also been reported (Table 2).^51^ For instance, in addition to its reported prognostic and predictive values, studies have shown that the serial detection of MGMT promoter methylation abnormalities in plasma or serum cfDNA from patients with gliomas was associated with tumor burden after treatment and with progression^14,52,53^

## Application of cfDNA Methylation-Based Machine-Learning Algorithms cfDNA

**Figure 1.**
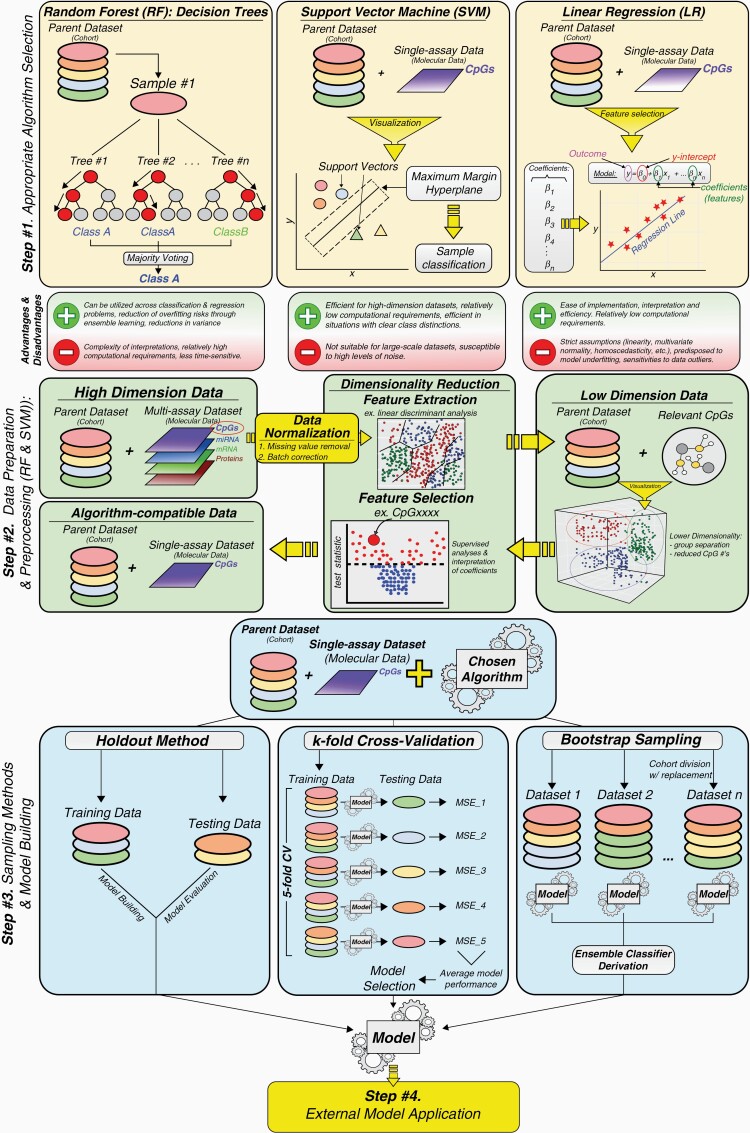
Schematic Outline of Conventional Steps to Developing Machine-Learning-based Models.

The application of computational methods, particularly machine-learning (Figure 1), to analyze the large array of molecular information generated through high-throughput omics data, constitutes a robust approach towards identification of valuable biomarkers for tumor diagnosis and prognostication and requires specialized algorithms, in addition to the ones used for tumor tissue, for liquid biopsy specimens. For instance, based on DNA methylation data obtained through targeted or genome-wide profiling of tumor-derived tissue or liquid biopsy specimens, studies reported on machine learning algorithms able to estimate tumor load, cell-of-origin, molecular subtypes, or prognosis (Table 3).^12,13,19,22,54–56^ Specifically to gliomas, machine learning models using cfDNA epigenetic markers profiled in blood (serum or plasma) or urine specimens showed greater than 80% accuracy in diagnosing these tumors, recapitulating the diagnostic accuracy obtained using tissue-derived methylation markers from glioma.^12,42,57^

## Final Remarks

Here we summarize the results of studies that used minimally or noninvasive liquid biopsy approaches to detect methylation-based markers to diagnose and prognosticate gliomas and explore the feasibility of some methods to monitor tumor evolution and treatment in real time. Although promising, there remains a need for standardized operating procedures involving the pre-analytical factors (biosource type, molecular profiling technologies, and data analysis strategies) as well as validation in larger cohorts and prospective designed studies before moving the application of this approach into clinical practice.^11,14,58^ Ongoing advancements in technologies and strategies involved in the isolation, detection and data analysis have shown improvements in the identification of relevant methylation markers using the minute amounts of cellular and molecular elements released by CNS tumors and in reproducibility of DNA methylation-based cfDNA analysis. Some companies are also developing assays that simultaneously assess different combinations of analytes in ctDNA such as fragmentomics and nucleosome positioning, next-generation sequencing, and immunoassays.^11,14,58^

Consortia in the liquid biopsy field such as the “Brain-Liquid Biopsy consortium”, established in 2020, has the potential to mirror the achievements of The Cancer Genome Atlas (TCGA) and ongoing Glioma Longitudinal AnalySiS (GLASS) consortia^10^ facilitating the standardization of procedures and the generation of reliable molecular and imaging data from large cohorts of patients.

## Future Perspectives

Development of a user-friendly web-based platform to upload molecular and clinical data extracted from liquid biopsy specimens followed by automatic normalization, random forest classification using methylation markers, and PDF report generation regarding tumor subtyping and prognosis is crucial and has the potential to mirror the success of web platforms developed for tumor tissue analysis,^20^ This proposed platform will allow sharing data and facilitate fast and secure communication between researchers, physicians, and patients. Global access to this web-based platform will further expand the number of patients profiled and accelerate the efforts towards its validation. The success of these efforts will ultimately result in the much needed enhancement in treatment opportunities and improvement of quality of life for patients with brain tumors.
